# Preoperative education with image illustrations enhances the effect of tetracaine mucilage in alleviating postoperative catheter-related bladder discomfort: a prospective, randomized, controlled study

**DOI:** 10.1186/s12871-018-0653-y

**Published:** 2018-12-22

**Authors:** Li Zhou, Le Zhou, Leilei Tian, Daojun Zhu, Ziwen Chen, Chang Zheng, Ting Zhou, Xianzheng Zeng, Xiaojuan Jiang, Chunling Jiang, Lulong Bo

**Affiliations:** 1 Department of Anaesthesiology and Translational Neuroscience Center, West China Hospital, Sichuan University, Chengdu, 610041 Sichuan China; 2 Department of Anaesthesiology, Sichuan Jinxin Women and Children’s Hospital, Chengdu, 610011 Sichuan China; 3 Faculty of Anaesthesiology, Changhai Hospital, Naval Medical University, Shanghai, 200433 China

**Keywords:** Catheter-related bladder discomfort, Urethral catheterization, Preoperative education, Image illustration, Tetracaine

## Abstract

**Background:**

Catheter-related bladder discomfort (CRBD), secondary to catheterization of urinary bladder is distressing. The aim of this study was to assess the efficacy of preoperative education on CRBD with image illustration for alleviating CRBD.

**Methods:**

Sixty adult male patients, undergoing elective colonal and rectal surgery, were randomized to receive tetracaine mucilage instilled into the urethra and applied to the catheter (tetracain group), or receive tetracaine mucilage in combination with image illustration on CRBD (image group) before urethral catheterization. The incidence and severity of CRBD were assessed at 0.5, 1, 2, and 6 h after patients’ extubation. The severity of postoperative pain, incidence of postoperative agitation and other adverse events were also recorded.

**Results:**

Patients in image group reported remarkably less CRBD than those in tetracaine group at 0.5,1, 2 and 6 h after extubation (20, 20, 6.7 and 6.7% *v.s.* 60, 73.3, 53.3 and 53.3%, respectively, *P*<0.01). Severe CRBD was not reported in either group. However, the incidence of moderate CRBD was significantly lower in image group, with 6.7% at 1 h and thereafter none occurred, compared to 6.7% at 0.5 h, and increasing to 20% at 1 h, 2 h and 6 h in tetracaine group, respectively. Moreover, patients in image group suffered less moderate to severe postoperative pain than that of tetracaine group (13.3% *v.s.* 40.0% at 1 h, *P* = 0.039, 33.3% *v.s.* 60% at 2 h and 6 h, *P* = 0.038).

**Conclusions:**

Preoperative education on uretheral catheterization via image illustrations could enhance the effect of tetracaine mucilage in reducing both the incidence and severity of CRBD.

**Trial registration:**

The trial was registered at www,clinicaltrials.gov with registration number NCT03199105 (retrospectively registered). Date of trial registration which is “June 26, 2017”.

**Electronic supplementary material:**

The online version of this article (10.1186/s12871-018-0653-y) contains supplementary material, which is available to authorized users.

## Background

Catheter-related bladder discomfort (CRBD), characterized by a burning sensation with an urge to void or discomfort in the suprapubic area [1, 2], are extremely distressing, and are frequently associated with emergence agitation [3, 4], exacerbated postoperative pain and other postoperative complications [3, 5]. Systemic administration of tramadol, tolterodine, oxybutynin, or gabapentin, et al., were reported effective in reducing CRBD [2, 3, 6–10]. Nevertheless, side effects such as postoperative nausea, vomiting, sedation, and dry mouth, et al., usually occur [2, 6, 7, 11]. Lubricants containing local anesthetic such as lidocaine or tetracaine [12, 13], instilled into the urethra or applied on the catheter, are increasingly used in urethral catheterization to minimize urethral trauma and CRBD to certain extent [1]. However, no effective treatment for CRBD without adverse effects has been established yet.

On the other hand, growing evidence shows that preoperative anxiety contributes greatly to postoperative pain and discomfort [14]. Studies in patients have demonstrated that specific education, via verbal instruction and/or in conjunction with pamphlet, photo file or illustration, given prior to the surgery [15] can help patients obtain better pain relief [16, 17], alleviate anxiety and discomfort [18, 19]. However, for patients with low literacy skills, preoperative education via pictures and illustrations are especially useful [20]. These findings shed light on the possibility that specific preoperative education on uretheral catheterization may decrease postoperative CRBD.

Giving specific information via verbal instruction in conjunction with image illustration to patients requiring urethral catheterization, our aim is to determine whether preoperative education on uretheral catheterization enhances the effect of local anesthetic tetracaine mucilage in alleviating postoperative CRBD.

## Methods

After obtaining Ethics approval (Ethical Committee N° 2014–159) from the Ethical Committee of West China Hospital, Chengdu, China on 23 December 2014, we conducted this prospective, randomized, controlled study in West China hospital according to the principles expressed in the Declaration of Helsinki. From July 2017 to May 2018, Adult, male patients (18–75 yr), with an ASA physical status I or II, who were to undergo elective colonal and rectal surgery with surgical duration of at least 2 h, requiring catheterization of the urinary bladder and were able to understand the rationale of the study and provided with informed consent, were consecutively included. Patients with a bladder outflow obstruction, e.g.*,* continuous feeling of a full bladder, frequent urination, pain during urination (dysuria), problems starting urination (urinary hesitancy), slow, uneven urine flow, at times being unable to urinate, et al., or a medical history of bladder outflow obstruction, which is diagnosed by urologist, overactive bladder (frequency > 3 times in the night or > 8 times in 24 h), neurogenic bladder, end-stage renal disease (urine output < 500 mL/24 h), coagulopathy, morbid obesity, disturbance of central nervous system, chronic pain, chronic medicine usage (e.g., opioids, anticholinergic or NSAID), and any psychiatric disease were excluded from the study.

**Fig. 1:**
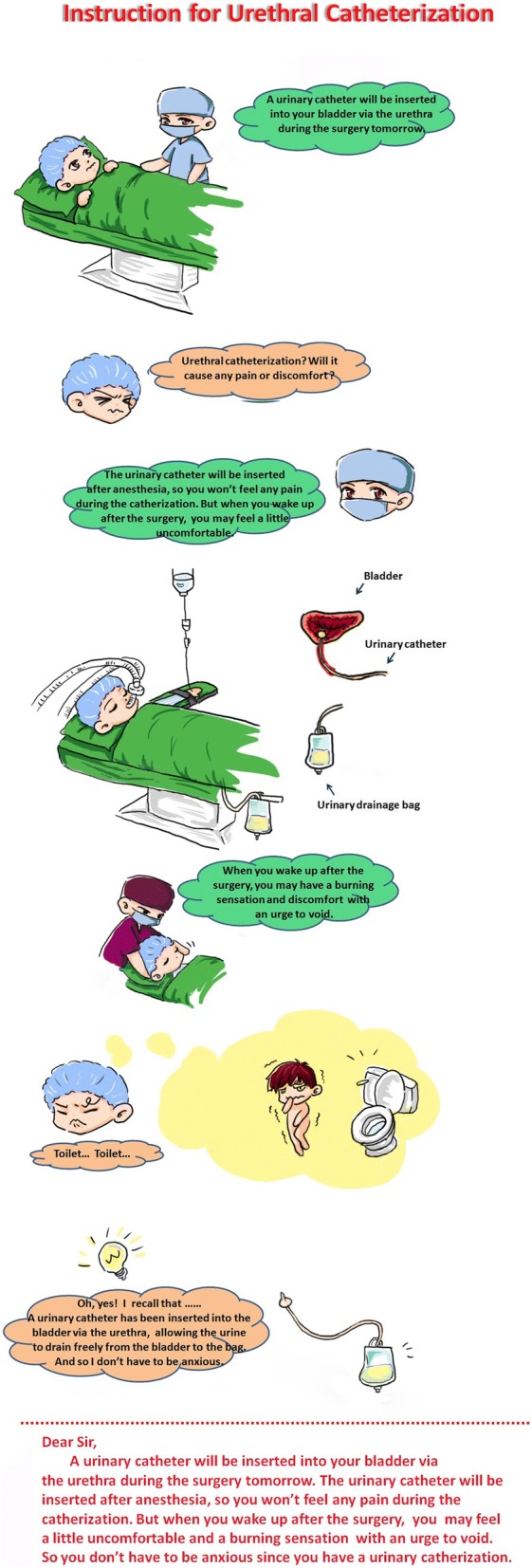
Instruction for uretheral catherization

Patients enrolled were randomized to either group using the computer-generated random numbers. A sealed envelope containing a random number was opened during the preoperative interview (one day prior to surgery) by a nurse. Patients in tetracaine group received 1% tetracaine mucilage (Xi’an Lijun Pharmaceutical Co., Ltd., Xi’an, China), instilled into the urethra and applied to the catheter before urethral catheterization after the anesthesia induction. The patients in image group were educated on the whole process from catheterization to emergence and the symptoms of CRBD by image illustration (Fig. 1) during the preoperative interview. Then the urethral catheter was prepared and placed in the same way as in tetracain group.

All participants received a peripheral intravenous line using a 16-gauge catheter, and routine anesthesia monitoring including electrocardiography, heart rate, noninvasive blood pressure, and pulse oximetry continuously after arriving at operation room. Anesthesia was induced with midazolam 0.03 mg·kg^− 1^, sufentanil 0.4 μg·kg^− 1^, cis-atracurium 0.2 mg·kg^− 1^, and propofol 2 mg·kg^− 1^. Anesthesia was maintained with inhalation of 1.5–2.5% vol of sevoflurane and intermittent sulfentanil and cis-atracurium as required. Urinary catheterization was performed with a 16 Fr Foley catheter and the balloon was inflated with 10 ml of 0.9% NaCl, then fixed with an adhesive tape without any traction and allowed the urine to drain freely into a bag. Last dose of sulfentanil was administered 30 min before completion of surgery. After the surgery, muscle relaxation was antagonized with neostigmine 0.02 mg·kg^− 1^ and glycopyrrolate 0.01 mg·kg^− 1^. Patients were then extubated and transferred to the postanesthesia care unit (PACU). If patients complained about pain in the PACU, they were enquired to confirm whether it was incision pain or discomfort related to urinary bladder. Sufentanil 5 μg *i.v.*was given to patients with VAS scores≥4 after confirmation of postoperative pain but not for CRBD.

The primary outcome of the study is the incidence of CRBD. The secondary outcomes include the severity of CRBD, the incidence and severity of postoperative pain, the incidence of postoperative agitation, and adverse events, such as respiratory depression (SpO_2_ < 90%), deep sedation, and toxicity of tetracaine. All of these outcomes were assessed at 0.5, 1, 2, and 6 h after patients’ extubation by a nurse blinded to the study design in PACU. The number of patients requiring sufentanil as rescue analgesics in PACU was also recorded.

CRBD was defined as a burning sensation at urethra with an urge to void, urinary frequency and painful discomfort in the supra-pubic region. The severity of CRBD was assessed according to the following scaling system: no CRBD; mild CRBD (complaint about CRBD on questioning only); moderate CRBD (complaint about CRBD without enquiring); and severe CRBD (complaint about CRBD without enquiring, with urinary urgency demonstrated by a spontaneous behavioral response such as flailing limb, verbal responses, or attempt to remove the catheter) [21]. Postoperative pain was assessed using a visual analog scale (VAS) score of 0–10, where 0 represents no pain and 10 represents worst imaginable pain. Postoperative agitation and sedation levels were determined by Riker Sedation-Agitation Scale [22], where 5 to 7 represents agitation and 1 to 3 represents deep sedation (Additional file 1: Table S1).

### Sample size and statistical analysis

In our pilot study, the incidence of CRBD was 55% in tetracain group. We assumed that preoperative education with image illustration would reduce the incidence of CRBD to 20%. Based on a statistical power of 80% with a two-sided level of significance of 5%, a sample size of 58 subjects were required (29 per group). A sample size of 64 subjects (32 per group) were planned to account for an expected dropout of 10%.

All data analysis was performed using SPSS software (version 22.0, SPSS Inc., Chicago, Illinois). The normality of continuous data was tested by one-sample Kolmogorov-Smirnov test. We showed the mean (standard deviation) for a continuous data set if its normality assumption is met. Otherwise, we showed the median (interquartile range) for the data set. For continuous data sets that meet normality assumption, their population means were compared by independent samples Student’s test. The population proportions of categorical data sets were compared using chi-square test or Fisher’s exact test or Rank sum test. *P*-value < 0.05 was considered significant.

## Results

**Fig. 2:**
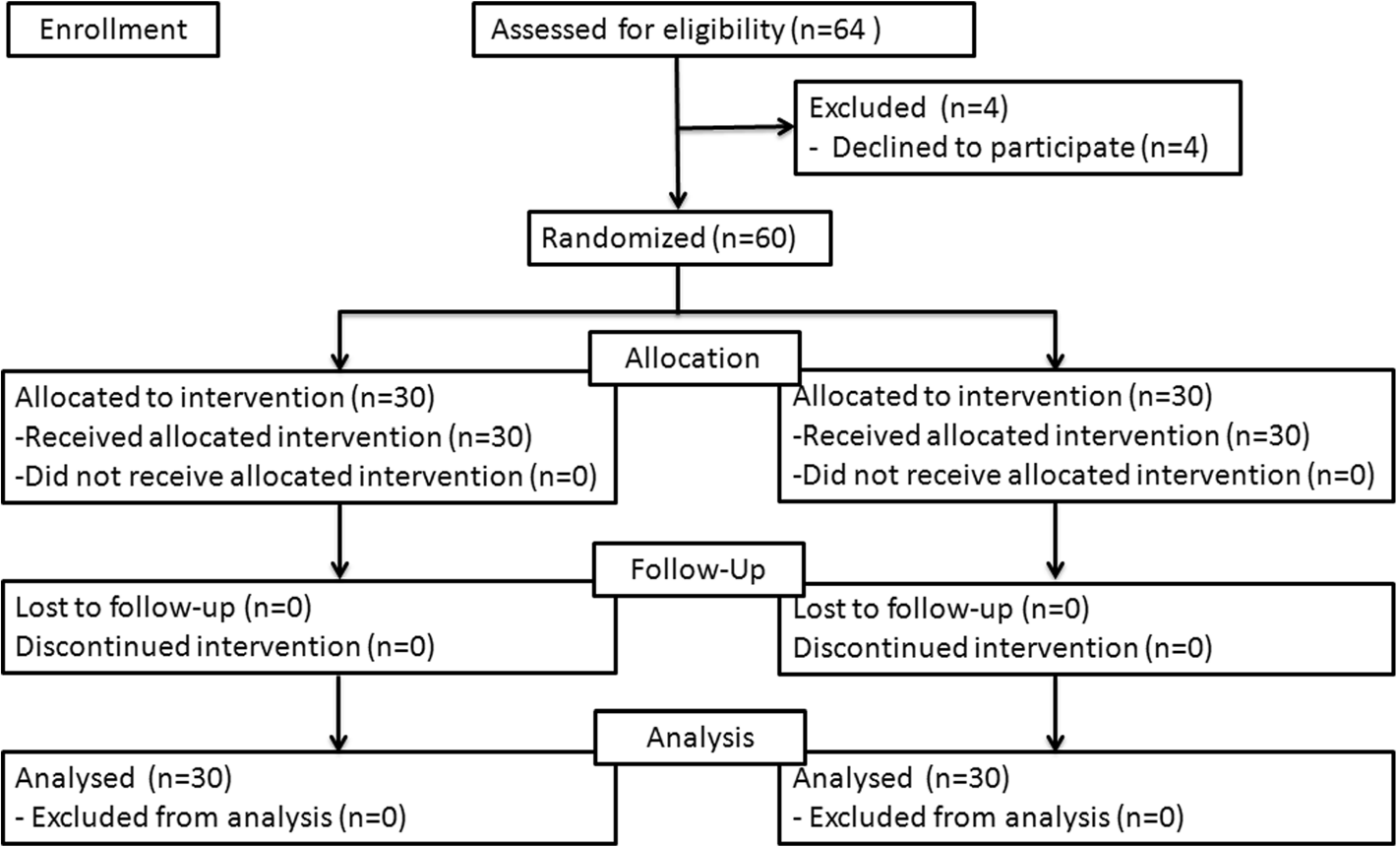
CONSORT diagram

Among the 64 patients who were eligible for the study, 4 patients were excluded for that they refused to participate in the study (Fig. 2). Of the remaining 60 patients, 30 were randomized to tetracaine group and 30 were randomized to image group (Fig. 2). And the data of sixty remaining patients were analysed.

Patients’ baseline characteristics, surgical duration and the doses of intraoperative analgesics were similar between two groups (Table 1).

**Table 1:** Baseline characteristics of patients. Values are given as mean ± SD or number of patients (%)

	Tetracaine group (*n* = 30)	Image group (n = 30)	*P* value
Age(yr)	58.14 ± 10.08	51.27 ± 13.58	0.136
Weight(Kg)	59.13 ± 9.63	62.13 ± 8.78	0.380
Height(cm)	166.38 ± 5.04	167.92 ± 6.55	0.509
Education level			0.177
Illiteracy	2 (6.7%)	0 (0%)	
Primary school	6 (20.0%)	12 (40.0%)	
Middle school	12 (40.0%)	14 (6.7%)	
High school	4 (13.3%)	0 (0%)	
College and above	6 (20.0%)	4 (13.3%)	
Duration of operation(h)	3.13 ± 1.13	2.71 ± 0.82	0.334
Intraoperative sufentanyl(ug/kg)	0.43 ± 0.30	0.41 ± 0.26	0.842

Patients in image group reported remarkably less CRBD than those in tetracaine group at 0.5 h (20% *v.s.*60%, *P* = 0.002), 1 h (20% *v.s.*73.3%, *P* = 0.000), 2 h (6.7% *v.s.* 53.3%, *P* = 0.000), and 6 h (6.7% *v.s.*53.3%, *P* = 0.000) after extubation, respectively (Table 2). Moreover, the severity of CRBD observed were significantly different between two groups. Although none of the patients in either group reported severe CRBD, the incidence of moderate CRBD was remarkably lower in image group, with only 6.7% (2/30) at 1 h and thereafter none occurred in image group, whereas compared to 6.7% (0.5 h), and increasing to 20% at1 h, 2 h and 6 h in tetracaine group, respectively (Table 3), These data suggest that image illustration enhanced the effect of tetracaine in decreasing both the incidence and severity of CRBD.

**Table 2:** Incidence of catheter-related bladder discomfort. Values are given as number of patients (%)

	Tetracaine group (n = 30)	Image group (n = 30)	P value
0.5 h	18 (60.0%)	6 (20.0%)	0.002
1 h	22 (73.3%)	6 (20.0%)	0.000
2 h	16 (53.3%)	2 (6.7%)	0.000
6 h	16 (53.3%)	2 (6.7%)	0.000

**Table 3:** Severity of catheter-related bladder discomfort. Values are given as number of patients (%)

	Tetracaine group (n = 30)			Image group (*n* = 30)			P value
	Mild	Moderate	Severe	Mild	Moderate	Severe	
0.5 h	16(53.3%)	2(6.7%)	0(0%)	6 (20.0%)	0 (0%)	0 (0%)	0.003
1 h	16 (53.3%)	6 (20.0%)	0 (0%)	4 (13.3%)	2 (6.7%)	0(0%)	0.000
2 h	10 (33.3%)	6 (20.0%)	0 (0%)	2 (6.7%)	0 (0%)	0 (0%)	0.000
6 h	10 (33.3%)	6 (20.0%)	0 (0%)	2 (6.7%)	0 (0%)	0 (0%)	0.000

Unexpectedly, there was no significant difference in incidence of postoperative pain (assessed by VAS scores) over time between two groups. However, stratified analysis showed that the occurrence of moderate to severe pain (VAS score ≥ 4) were lower in image group than that of tetracaine group (13.3% *v.s.* 40% at 1 h, *P* = 0.039; 33.3% *v.s.* 60% at 2 h, *P* = 0.038; 33.3% *v.s.* 60% at 6 h, *P* = 0.038, respectively) (Table 4). In line with these findings, rescue analgesics (sufentanil was given if VAS ≥ 4) were required more often in the tetracaine group than in the image group (40% *v.s.* 13.3% at 1 h, *P* = 0.039; 60% *v.s.* 33.3% at 2 h, *P* = 0.038; 60% *v.s.* 33.3% at 6 h, *P* = 0.038, respectively). However, the average dose of sufentanil in each patient receiving rescue sufentanil showed no difference (0.49 ± 0.27 *v.s.* 0.41 ± 0.26 μg/kg, *P* = 0.352) (Additional file 2: Table S2, Additional file 3: Table S3).

**Table 4:** Incidence and severity of postoperative pain

	Tetracaine group (n = 30)		Image group (n = 30)		P value
	Mild	Moderate & Severe	Mild	Moderate & Severe	
0.5 h	26(86.7%)	4(13.3%)	28 (93.3%)	2 (6.7%)	0.671
1 h	18 (60.0%)	12 (40.0%)	26 (86.7%)	4 (13.3%)	0.039
2 h	12 (40.0%)	18 (60.0%)	20 (66.7%)	10 (33.3%)	0.038
6 h	12 (40.0%)	18 (60.0%)	20 (66.7%)	10 (33.3%)	0.038

Interestingly, patients in image group did not have agitation (defined as SAS score 5 to7) after extubation, whereas the incidences of agitation in tetracaine group were 6.7% at 1 h, which increased to 16.7% at 2 h and 13.3% at 6 h, respectively (Table 5). The overall status of sedation level for all the patients showed no difference between 2 groups (Additional file 4: Table S4).

**Table 5:** Incidences of postoperative agitation

	Tetracaine group (n = 30)	Image group (n = 30)	P value
0.5 h	0 (0%)	0 (0%)	–
1 h	2 (6.7%)	0 (0%)	0.492
2 h	5 (16.7%)	0 (0%)	0.052
6 h	4 (13.3%)	0 (0%)	0.112

Furthermore, no patients suffered from toxicity of local anaesthetics and other adverse events, including respiratory depression, and deep sedation in both groups.

## Discussion

The present study demonstrates that preoperative education on uretheral catheterization and CRBD via verbal instruction in conjunction with image illustrations could enhance the effect of tetracaine mucilage in reducing both the incidence and severity of CRBD and alleviating emergence agitation.

CRBD is a common complication after uretheral catheterization in surgical male patients [6], with an incidence ranging from 47 to 90% [3, 23]. It is frequently associated with emergence agitation [3, 4], exacerbated postoperative pain, and other postoperative complications [3, 5]. Lubricants containing local anesthetic such as lidocaine or tetracaine, instilled into the urethra or applied to the catheter, are commonly used in urethral catheterization to minimize urethral trauma and decrease the incidence of postoperative CRBD [1] to certain extent. However, effective prevention and treatment for CRBD without adverse effects, has not been established yet.

On the other hand, there is growing evidence indicating that preoperative education programs could be implemented to alleviate anxiety, postoperative pain and discomfort [14]. These programs, with content varies greatly, may include verbal instruction, one or combination of an operating area visit, and peer modelling preparation using videos, instruments or images [18]. In our study, in addition to applying lubricants containing tetracaine during urethral catheterization, we used verbal instruction combining with image illustration during preoperative visit,explaining the whole process from urethral catheterization and the symptoms of CRBD, which will enable the patients to understand and remember the related information. We found that preoperative education via verbal instruction in conjunction with image illustrations enhanced the effect of tetracaine mucilage by reducing at least a half of the incidence of CRBD, and further decreases the severity of CRBD. These findings indicated that image illustration in combination with tetracain, as a simple and economic way, is effective in decreasing both the incidence and severity of CRBD.

Interestingly, patients in image group experienced less moderate and severe pain, unlike up to 40–60% in tetracaine group after 1 h. Moreover, fewer rescue analgesics were required in the image groups than that in the tetracaine group. This emphasizes the need for effective preoperative education on CRBD via such simple and economic way to be included in the preoperative preparation.

Several limitations of the current study should also be considered. Firstly, only male patients who were to undergo elective colonal and rectal surgery, were included. We did not include female patients and did not observe other procedures, such as urological surgery, because these surgeries may affect the occurrence of postoperative CRBD. Therefore, limitations exist in the application of image preoperative education to patients who undergo other operations requiring urethral catheterization. Secondly, urethral catheterization was performed using unified 16-Fr Foley catheters, and we did not compare Foley catheters with other size catheters. A large diameter Foley catheter is a known risk factor for CRBD. Thirdly, as most studies investigating CRBD suggested, their observation period of CRBD is usually within 6 h after operation [1, 2, 24, 25], partly because the incidence of CRBD reached its peak mainly in 6 h after surgery, and patients in this study usually stayed in PACU for 6 h, therefore, we did not evaluate whether image illustrations are beneficial for longer duration. Lastly, we did not measure the uroflowmetry, the residual urine and the size of prostate which might be a confounding factor to affect the result.

## Conclusions

In conclusion, preoperative education on uretheral catheterization via verbal instruction in conjunction with image illustrations could enhance the effect of tetracaine mucilage in reducing both the incidence and severity of CRBD. Moreover, the preoperative education reduced the incidence of postoperative from moderate to severe pain along with decreasing the number of patients requiring sufentanil, and alleviating emergence agitation. We therefore suggest that preoperative education via image illustration on uretheral catheterization should be included in the preoperative preparation for surgical patient requiring catheterization of the urinary bladder. Image illustration in combination with tetracaine is a simple, economic, safe and effective method to decrease both the incidence and severity of CRBD.

## Additional files

Additional file 1: **Table S1.** Riker Sedation-Agitation Scale. (DOCX 64 kb)

Additional file 2: **Table S2.** The number of patients received rescue sufentanil in each group. (DOCX 63 kb)

Additional file 3: **Table S3.** The average dose of sufentanil in each patient received rescue analgesics in the PACU. (DOCX 61 kb)

Additional file 4: **Table S4.** The status of sedation level. (DOCX 62 kb)

## Abbreviations

CRBD: Catheter-related bladder discomfort
PACU: Postanesthesia care unit
SAS: Sedation-Agitation Scale
VAS: Visual Analogue Scale/Score
