# Supplementary material for: Preoperative education with image illustrations enhances the effect of tetracaine mucilage in alleviating postoperative catheter-related bladder discomfort: a prospective, randomized, controlled study
Source: BMC Anesthesiol. 2018 Dec 22;18:204. doi: 10.1186/s12871-018-0653-y (PMC6303915; doi:10.1186/s12871-018-0653-y)
Supplement: Supplementary file 1 — Table S1. Riker Sedation-Agitation Scale. (DOCX 64 kb) [file 12871_2018_653_MOESM1_ESM.docx]

**Additional file 1: Table S1 Riker Sedation-Agitation Scale**

| Score | Term | Descriptor |
| --- | --- | --- |
| 7 | Dangerous Agitation | Pulling at ETT, trying to remove catheters, climbing over bedrail, striking at staff, trashing side-to-side |
| 6 | Very Agitated | Requiring restraint and frequent verbal reminding of limits, biting ETT |
| 5 | Agitated | Anxious or physically agitated, calms to verbal instructions |
| 4 | Calm and Cooperative | Calm, easily arousable, follows commands |
| 3 | Sedated | Difficult to arouse but awakens to verbal stimuli or gentle shaking, follows simple commands but drifts off again |
| 2 | Very Sedated | Arouses to physical stimuli but does not communicate or follow commands, may move spontaneously |
| 1 | Unarousable | Minimal or no response to noxious stimuli, does not communicate or follow commands |
