# Supplementary material for: Preoperative education with image illustrations enhances the effect of tetracaine mucilage in alleviating postoperative catheter-related bladder discomfort: a prospective, randomized, controlled study
Source: BMC Anesthesiol. 2018 Dec 22;18:204. doi: 10.1186/s12871-018-0653-y (PMC6303915; doi:10.1186/s12871-018-0653-y)
Supplement: Supplementary file 2 — Table S2. The number of patients received rescue sufentanil in each group. (DOCX 63 kb) [file 12871_2018_653_MOESM2_ESM.docx]

**Additional file 2: Table S2 The number of patients received rescue sufentanil in each group.**

|  | Tetracaine group (n=30) | Image group (n=30) | | | P value |  |
| --- | --- | --- | --- | --- | --- | --- |
| 0.5h | 4（13.3%） | | 2(6.7%) | 0.671 | | |
| 1h | 12(40.0%) | | 4(13.3%) | 0.039 | | |
| 2h | 18(60.0%) | | 10(33.3%) | 0.038 | | |
| 6h | 18(60.0%) | | 10(33.3%) | 0.038 | | |
