# Supplementary material for: Preoperative education with image illustrations enhances the effect of tetracaine mucilage in alleviating postoperative catheter-related bladder discomfort: a prospective, randomized, controlled study
Source: BMC Anesthesiol. 2018 Dec 22;18:204. doi: 10.1186/s12871-018-0653-y (PMC6303915; doi:10.1186/s12871-018-0653-y)
Supplement: Supplementary file 3 — Table S3. The average dose of sufentanil in each patient received rescue analgesics in the PACU. (DOCX 61 kb) [file 12871_2018_653_MOESM3_ESM.docx]

**Additional file 3: Table S3 The average dose of sufentanil in each patient received rescue analgesics in the PACU**

| Average dose of sufentanil （ug/kg） | Tetracaine group (n=30) | Image group (n=30) | P value | |
| --- | --- | --- | --- | --- |
| within 6h | 0.49±0.27 | 0.41±0.26 | | 0.352 |
