# Supplementary material for: Preoperative education with image illustrations enhances the effect of tetracaine mucilage in alleviating postoperative catheter-related bladder discomfort: a prospective, randomized, controlled study
Source: BMC Anesthesiol. 2018 Dec 22;18:204. doi: 10.1186/s12871-018-0653-y (PMC6303915; doi:10.1186/s12871-018-0653-y)
Supplement: Supplementary file 4 — Table S4. The status of sedation level. (DOCX 62 kb) [file 12871_2018_653_MOESM4_ESM.docx]

**Additional file 4: Table S4 The status of sedation level**

| SAS score | Tetracaine group (n=30) | Image group (n=30) | P value |
| --- | --- | --- | --- |
| 0.5h | 3.65±0.67 | 3.60±0.63 | 0.824 |
| 1h | 4.05±0.22 | 3.93±0.25 | 0.162 |
| 2h | 4.35±0.81 | 3.93±0.25 | 0.065 |
| 6h | 4.10±0.96 | 3.87±0.51 | 0.404 |
